# Control of Particle Size in Flame Spray Pyrolysis of Tb–doped Y_2_O_3_ for Bio-Imaging

**DOI:** 10.3390/ma13132987

**Published:** 2020-07-04

**Authors:** Sovann Khan, Yunseok Choi, Hak-Young Ahn, Jae Hyun Han, Byeong-Kwon Ju, Jaewon Chung, So-Hye Cho

**Affiliations:** 1 Photocatalysis International Research Center, Tokyo University of Science, 2641 Yamazaki, Noda-shi, Chiba 278-8510, Japan; sovannkhan@gmail.com; 2 Materials Architecturing Research Center, Korea Institute of Science & Technology, 5 Hwarang-ro 14-gil, Seongbuk-gu, Seoul 02792, Korea; yschoijx@gmail.com (Y.C.); hyahn@kist.re.kr (H.-Y.A.); han_2409@kist.re.kr (J.H.H.); 3 Department of Mechanical Engineering, Korea University, Anam-dong 5-ga, Seongbuk-gu, Seoul 02841, Korea; 4 Display and Nanosystem Laboratory, School of Electrical Engineering, Korea University, 145, Anam-ro, Seongbuk-gu, Seoul 02841, Korea; bkju@korea.ac.kr; 5 KIST School, Korea University of Science and Technology, 217 Gajeong-ro Yuseong-gu, Daejeon 34113, Korea

**Keywords:** Tb–doped Y_2_O_3_, flame spray pyrolysis, nanophosphor, bio-image, salt-assisted FSP, luminescence

## Abstract

Recently, the use of oxide-based nanomaterials for bio-imaging has received great attention owing to their remarkable stabilities as compared to those of conventional organic dyes. Therefore, the development of scalable methods for highly luminescent oxide materials with fine control of size has become crucial. In this study, we suggested modified flame spray pyrolysis (FSP) as a scalable method to produce a green-light emitting phosphor—Tb–doped Y_2_O_3_—in the nanometer size range. In our FSP method, an alkali salt (NaNO_3_) was found to be highly effective as a size-controlling agent when it is simply mixed with other metal nitrate precursors. The FSP of the mixture solution resulted in oxide composites of Y_2_O_3_:Tb^3+^ and Na_x_O. However, the sodium by-product was easily removed by washing with water. This salt-assisted FSP produced nano-sized and well-dispersed Y_2_O_3_:Tb^3+^ nanoparticles; their crystallinity and luminescence were higher than those of the bulk product made without the addition of the alkali salt. The nanoparticle surface was further coated with silica for biocompatibility and functionalized with amino groups for the attachment of biological molecules.

## 1. Introduction

Nanomaterials have been widely studied for bio-imaging owing to their unique physical and optical properties, such as large surface-area-to-volume ratio and quantum confinement effects [1]. Although various functional nanomaterials have been applied for bio-imaging, fluorescence imaging involves the use of luminescent nanoparticles [2]. Conventionally, organic fluorophores, which emit a visible light color of an appropriate wavelength upon irradiation, have been used for fluorescence imaging. However, their low stability, often resulting in photobleaching during bio-imaging, has led to the development of more robust inorganic nanomaterials [2]. In particular, oxide-based luminescent nanoparticles have recently gained attention as they have been proven as stable substitutes of the organic fluorophores [3,4]. Lanthanide-doped yttrium oxide is an excellent candidate for usage as luminescent nanoparticles for bio-imaging owing to its strong luminescence, high chemical and physical stabilities, and non-toxicity toward living cells [5,6,7].

A number of Y_2_O_3_-based phosphors were developed with a wide spectrum of strong visible light luminescence, such as red-light emitting Eu^3+^–doped Y_2_O_3_ phosphor, green-light emitting Tb^3+^–doped Y_2_O_3_, and blue-light emitting Tm^3+^–doped Y_2_O_3_, and they have been commercially used as color-conversion materials for lighting and display [8,9]. However, they have received limited success in bio-imaging until recent years [2,5] because their sizes are typically large (several microns). The conventional methods to produce Y_2_O_3_-based phosphors are solid-state reactions or wet chemical methods followed by high temperature post-annealing, and these methods often lead to large particle sizes and aggregations [10,11]. For bio-imaging, the sizes of luminescent materials need to be controlled such that they are small enough to be intergrated into living cells [1]. In addition, the aggregation of particles should be avoided for bio-compatibility. Hence, the control of both particle sizes and dispersity in an aqueous solution is essential for the bio-imaging application of the luminescent materials [4].

Flame spray pyrolysis (FSP) is one of the promising routes for the rapid and consecutive synthesis of various oxide materials from single to complex components [12,13]. Many attempts were made to synthesize Y_2_O_3_-based phosphors using FSP, and these phosphors exhibited superior luminescence properties than those obtained using conventional methods owing to their high crystallinity and homogenous dopant distribution. However, FSP commonly resulted in micron-size particles with a broad size distribution owing to an inhomogeneous temperature profile of the flame, which results in various degrees of aggregation and the agglomeration of primary particles [14,15]. To solve this problem, modified FSP processes have been studied in an effort to prevent aggregation and particle size growth [2,15,16].

In our previous study, we also successfully demonstrated the size control of Tb^3+^–doped Y_2_O_3_ phosphors using FSP by manipulations of liquid precursor solutions for spray, such as solvent dilution and the addition of organic additives [17,18]. However, using such methods achieved only limited success, as the resulting nanophosphors exhibited reduced luminescence as compared to that of the bulk products made using FSP. Although it is relatively common that the size reduction of phosphor materials resulted in a decrease in luminescence [19,20], our previous methods were not adaptable for synthesizing nanophosphors for bio-imaging owing to the low luminescence. 

Alkali salts (Na, K, Li) have been used as additives for enhancing the crystallinity of phosphor materials in many different synthetic methods, such as solid-state reactions, combustion, and pyrolysis [21,22,23,24]. In general, these salts serve as a molten medium (called as molten salts) for solid reactants during high-temperature reactions, which enables a facile transport of reactants to attain their highly crystalline state within a relatively short time [22]. The molten salts also help in preventing the aggregation of reactant particles as they cover and stabilize the surface of the particles [22,25]. Several researchers have also added alkali salts in FSP to produce lanthanide–doped Y_2_O_3_ phosphors [23,24]; however, they could not control the particle size to the submicron range, thus leaving scope for improvement.

In this report, we demonstrated alkali salt-added FSP as a successful method to produce Tb^3+^–doped Y_2_O_3_ phosphors that were approximately 100 nm in diameter with a narrow size distribution. The nanophosphors were single crystalline and highly luminescent, which made them suitable for bio-imaging. We were also able to coat the nanophosphor surface with a silica shell, which was then successfully functionalized with amine-terminated organic ligands for bio-applications. 

## 2. Experimental

### 2.1. Chemicals

Y(NO_3_)_3_∙6H_2_O and Tb(NO_3_)_3_∙6H_2_O were purchased from Alfa Aesar, Haverhill, MA, USA, and NaNO_3_ and 3-aminopropyltetraethoxysilane (APTES) were purchased from Sigma Aldrich, St. Louis, MI, USA. Ethanol (97%), tetraethoxyorthosilicate (TEOS), and ammonia solution (28% v/v in water) were purchased from Daejung Chem., Gyeonggi, Korea, and were used as received. Double-distilled water was used for an aqueous solution.

### 2.2. Alkali Salt-Added FSP of Tb^3+^-Doped Y_2_O_3_

**Figure 1:**
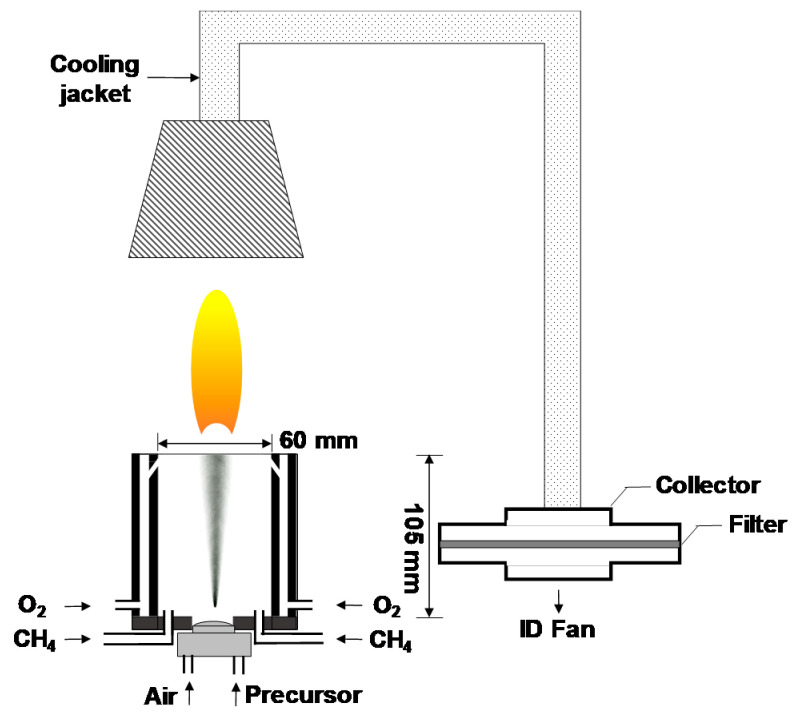
Experimental setup for flame spray pyrolysis (FSP).

The details of the FSP experimental setup are depicted in Figure 1, which is slightly modified from our previously reported setup [17,18]. The precursor solutions for FSP were prepared by dissolving metal nitrates in a mixture of ethanol and water solution. First, 9.9 μmol of Y(NO_3_)_3_∙6H_2_O, 0.1 μmol of Tb(NO_3_)_3_∙6H_2_O, and 0.1 mol of NaNO_3_ were dissolved into 50 mL of water via sonication for 20 min. The Tb doping concentration was adjusted to 1 mol% of Y (Tb/Y = 0.01) according to the results of our previous studies [17,18]. Further, 0.95 L of ethanol was added to the aqueous mixture, which was then stirred for 1 h at 25 °C. The mixture was sprayed using an atomizer (¼-air atomizing nozzle, Daewoo Precision Co., Gyeonggi, Korea) at a supply rate of 30 mL/min with a carrier gas of air at a flow rate of 6 L/min. The flame was generated with a constant supply of CH_4_ and O_2_ gases. The maximum temperature of the flame was measured using an R-type thermocouple, and the resident time of particles in the flame was less than 0.1 s. Flame-generated particles were cooled using a cooling jacket and collected using a metal mesh filter. Subsequently, the residual salts were removed with de-ionized water via water washing. Finally, the produced tint yellow powder was dried in an oven at 60 °C for 24 h. No further annealing was necessary.

### 2.3. Surface Functionalization of Tb^3+^-Doped Y_2_O_3_ Nanoparticles

Silica shell formation was conducted following the modified Stöber method [26]. First, 100 mg of Tb–doped Y_2_O_3_ nanoparticles that were synthesized with sodium salt and then washed with water were dispersed in ethanol (100 mL). After sonication for 30 min, 1.0 mL of TEOS was added into the ethanol solutions under vigorous stirring; then, 9 mL of water was dropped gradually, which was followed by the slow addition of 3 mL of ammonia solution. After 1 h of stirring, the colloidal solution was centrifuged to separate particles from the reaction mixture. The particles were washed with ethanol three times and dried in an oven at 60 °C. The formation of silica shell was confirmed by FT-IR (Fourier transform infrared) analysis. For further functionalization of the silica-coated Tb–doped Y_2_O_3_ nanoparticles with organic ligands, the silica-coated nanoparticles (100 mg) were dispersed in ethanol solution (100 mL) by sonication for 30 min, and then, 100 μL of APTES was added to the colloidal solution while stirring. To the mixture, 0.9 mL of water and 0.3 mL of ammonia solution were added slowly. After 1 h of stirring, the colloidal solution was centrifuged to separate particles from the reaction mixture, washed with ethanol three times, and dried in an oven to provide the amine-functionalized particles as a tint-yellow powder. The presence of organic ligands was evidenced by FT-IR and CHNS elemental analysis, indicating the successful coupling of APTES to the silica shell of Tb–doepd Y_2_O_3_ nanoparticles.

### 2.4. Characterizations

Particle morphology and crystallinity were studied using transmission electron microscopy (TEM, Talos F200X, FEI Company, Hillsboro, OR, USA), selected area electron diffraction (SAED, Talos F200X, FEI Company, Hillsboro, OR, USA), high-angle annular dark-field scanning transmission electron microscopy (HAADF-STEM, Talos F200X, FEI Company, Hillsboro, OR, USA), and field emission scanning electron microscopy (FESEM, Inspect F50, FEI Company, Hillsboro, OR, USA). Crystal structure analysis was performed using X-ray diffraction (XRD, D8 Advanced, Bruker Corporation, Billerica, MA, USA). Elemental mapping was carried out using energy dispersive X-ray spectroscopy (EDS, Talos F200X, FEI Company, Hillsboro, OR, USA). Hydrodynamic particle size was obtained using a dynamic light scattering (DLS) method by a particle size analyzer (Microtrac, NanotracBEL, Osaka, Japan). The optical properties such as photoluminescence (PL), photoluminescence excitation (PLE), and decay time of synthesized powder were studied using spectrophotometers (F-7000, Hitachi Inc., Tokyo, Japan). Fourier transform infrared (FT-IR) spectra were obtained by a transmittance mode (Platinum ATR, Bruker Corporation, Billerica, MA, USA). Elemental analysis was performed by X-ray fluorescence (XRF) (ZSX Primus II, Rigaku Corporation, Tokyo, Japan) for Tb, Y, Si, and a CHNS Analyzer (FLASH 2000, Thermo Scientific, Waltham, MA, USA) for C, H and N, elements. The zeta potentials of Y_2_O_3_ nanoparticles and surface-modified samples were analyzed by Zetasier Nano ZS (Malvern, Malvern, UK). For this analysis, each sample was suspended in a buffered solution of pH 7.4 (Sigma Aldrich, St. Louis, MI, USA).

## 3. Results and Discussion

**Figure 2:**
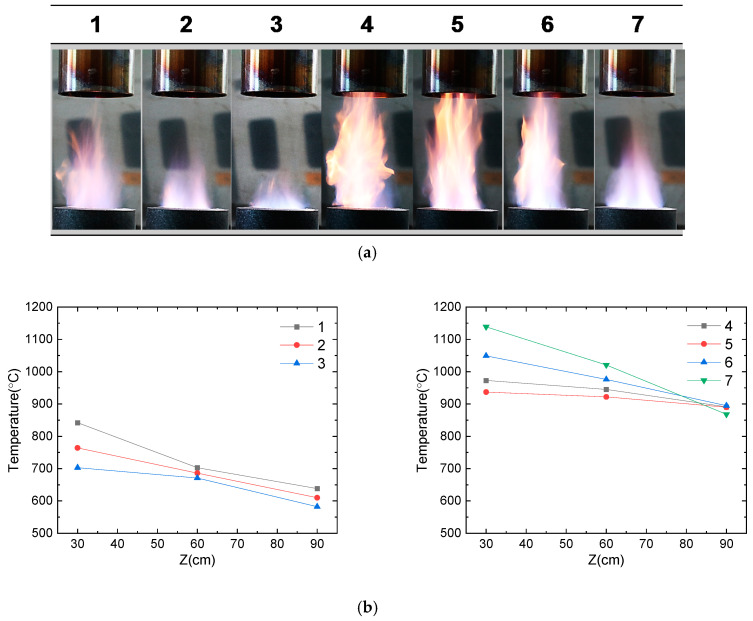
Flame characteristics with different ratios of fuel/oxidant gases (CH_4_/O_2_): (**a**) real-time images of flames and (**b**) temperature profile of each flame (#1–7) as a function of lengths (cm) from the flame burner.

For the optimization of the flame, the flow rates of CH_4_ and O_2_ gases were varied, and flame temperatures were monitored (Table 1). Camera images of the flame at different conditions and measured temperatures along the flame lengths from the burner top are presented in Figure 2. At a constant air flow rate of 6 L/min, the flame was longer with a higher CH_4_ flow rate (#1–3 vs. #4–7), while the supply of O_2_ gas resulted in the shortening of the flame. At a CH_4_ flow rate of 6 L/min (#1–3), the maximum temperatures of the flame decreased along with an increase in the O_2_ flow rate (850 °C, 775 °C, and 700 °C for #1, #2 and #3, respectively). However, at a higher CH_4_ flow rate of 9 L/min, the flame temperature increased along with an increase in the O_2_ flow rate (975 °C, 940 °C, 1050 °C, and 1150 °C for #4, #5, #6, and #7, respectively). Although the flame length of the condition of #7 was shorter than that of #5 and #6, the maximum temperature of the flame was highest for #7. Therefore, the flame condition of #7 is considered close to the complete combustion of CH_4_, and hence, it was selected for the nanoparticle synthesis.

**Figure 3:**
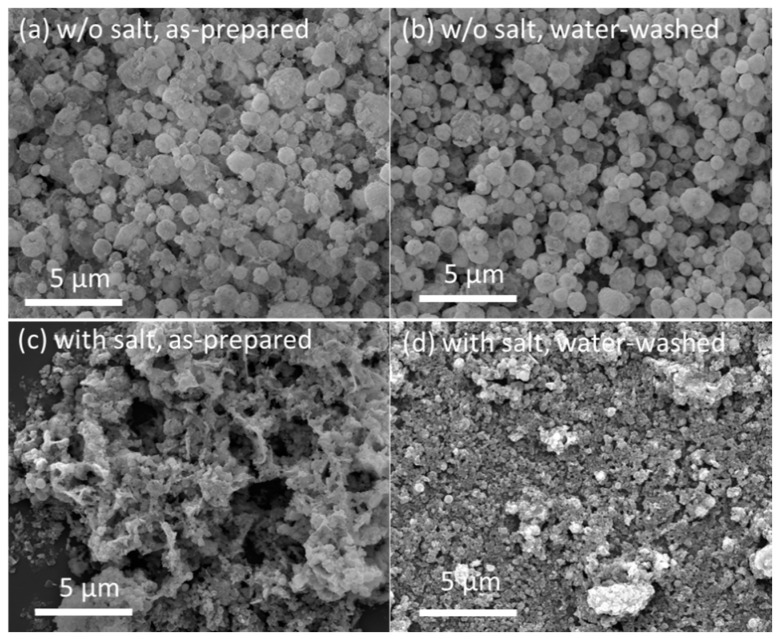
SEM images of: (**a**) as-prepared FSP product without sodium salts; (**b**) FSP product without sodium salts after washing with water; (**c**) as-prepared FSP product with sodium salts; and (**d**) FSP product with sodium salts after washing with water.

Next, FSP of Y_2_O_3_:Tb^3+^ with alkali salts ([Na]/[Y + Tb] = 10) was carried out at the selected flame condition of #7. The flame was stable during the FSP of the metal precursors, and the obtained powder was a tinted yellow color. For comparison, Y_2_O_3_:Tb^3+^ phosphors without the addition of alkali salts were also obtained at the same flame condition. Figure 3a,c show the SEM images of the FSP products synthesized without and with sodium salts, respectively. Hollow/shell-like spherical particles with broad size distribution (0.1–2 μm) were obtained using FSP without sodium salt addition (Figure 3a). In our previous report, we concluded that these hollow/shell-like spherical particles were formed owing to the preferential precipitation of precursors at the surface of droplets and their subsequent oxidation and aggregation/agglomeration at the outer shell [17]. The size and morphology of the FSP product without sodium salts were not altered after washing with water (Figure 3b). Upon the addition of sodium salts to the precursor solution, which is 10 times equivalent to the metal nitrate precursors of Y and Tb, the FSP resulted in the completely different size and morphology of Y_2_O_3_:Tb^3+^ (Figure 3c). Large spherical particles were not formed. However, submicron-sized particles were observed that appeared to be slightly connected as a sheet structure. When the particles were washed with water, well-dispersed fine particles appeared (Figure 3d). 

**Figure 4:**
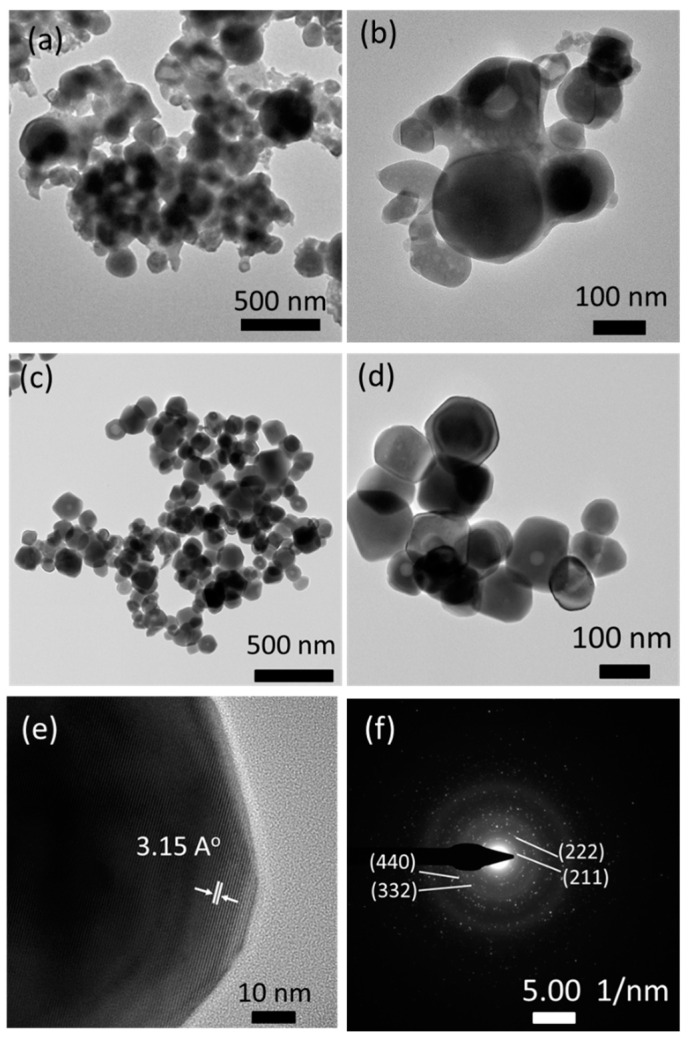
TEM images of FSP products prepared with sodium salts: (**a**,**b**) as-prepared; (**c**,**d**) after washing with water; (**e**) high resolution image; and (**f**) selected area electron diffraction (SAED) pattern.

The FSP product prepared with sodium salts was further examined using the TEM images (Figure 4). The particles appeared covered with amorphous phases before washing with water, which may be associated with residual sodium species (Figure 4a,b). However, the amorphous species were all removed upon washing with water; moreover, well-dispersed and highly crystalline particles of 50–200 nm diameter (Figure 4c,d) were observed. A high-resolution TEM image shown in Figure 4e indicates that the particles are single crystalline with a clear view of crystal lattice planes. A lattice distance of 3.15 Å is observed, which corresponds to the lattice plane (222) of the cubic phase Y_2_O_3_ according to JCPDS#71-0099. The SAED image in Figure 4f also indicates the high crystallinity of particles, and its dotted patterns exhibit (211), (222), (332), and (440) lattice planes of the cubic phase Y_2_O_3_. 

**Figure 5:**
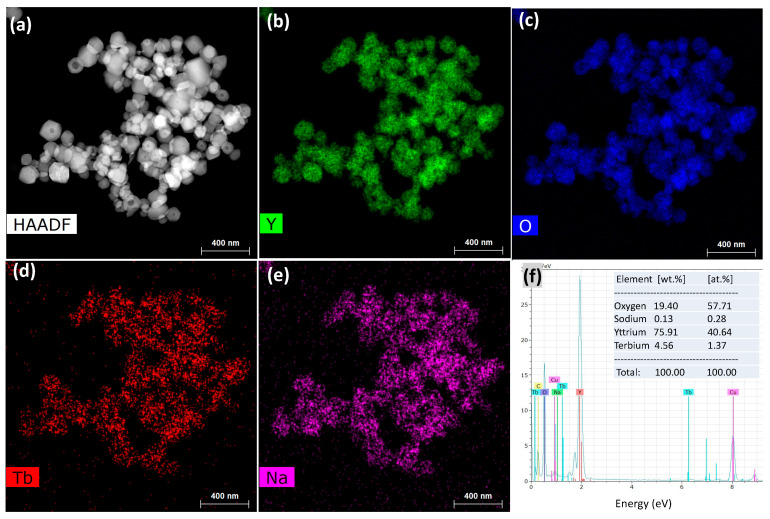
(**a**) High-angle annular dark-field scanning transmission electron microscopy (HAADF-STEM) image; (**b**–**e**) elemental mapping images of Y, O, Tb, and Na; and (**f**) energy-dispersive X-ray spectroscopy (EDS) spectrum and elemental compositions of FSP product prepared with sodium salts and water washing.

HAADF-STEM and elemental mapping images were also obtained to examine the elemental distribution in the particles (Figure 5). Tb ions were found to be well dispersed into Y_2_O_3_ without the formation of aggregates, indicating their homogeneous doping (Figure 5b–d). Remarkably, sodium was also found to be distributed over Y_2_O_3_ in spite of severe washing with water. The relative elemental composition was recorded as 40.64 at % of Y, 57.71 at % of O, 1.37 at % of Tb, and 0.28 at % of Na (Figure 5f and its inset) using EDS analysis; the presence of Na was attributed to its incorporation into the crystal lattice of Y_2_O_3_.

**Figure 6:**
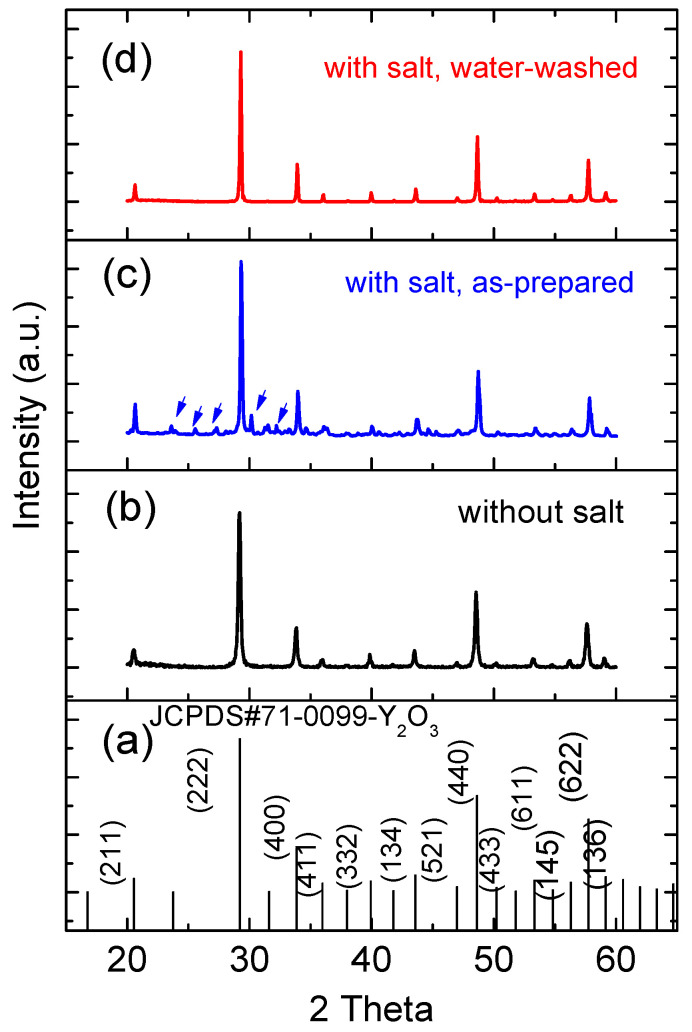
(**a**) JCPDS #71-0099 reference pattern of cubic phase Y_2_O_3_; XRD patterns of (**b**) as-prepared FSP product without sodium salts; (**c**) as-prepared FSP product with sodium salts; and (**d**) water-washed FSP product with sodium salts.

The XRD analysis of the FSP products prepared without and with sodium salts showed that all the products form Y_2_O_3_ in the cubic phase (Figure 6) and corresponded well with the observations made using TEM. No impurity peak associated with Tb was detected, indicating the homogenous doping of Tb into Y_2_O_3_ for all samples. However, Na-related impurity peaks (marked by arrows in Figure 6c) were observed in the as-prepared FSP products with sodium salts, which also correlated well with the above EDS analysis. The impurity peaks were matched to those of NaNO_3_ precursor and its oxidized product, Na_2_O. However, these impurities were soluble in water, and hence, they were removed by washing with water (Figure 6d). The full-width at half-maximum (FWHM) [27] of the XRD peaks at (222) of Y_2_O_3_ prepared without and with sodium salts were compared, and they were 0.245° and 0.139° respectively, indicating that the salt addition to FSP improved the crystallinity of the resulting products. 

**Figure 7:**
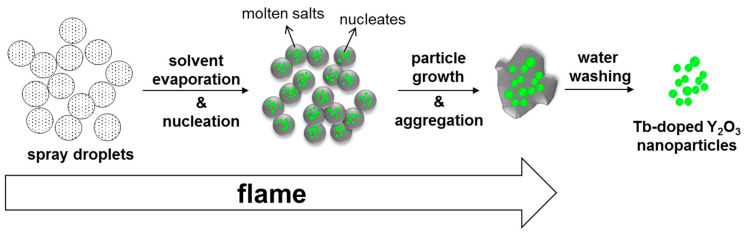
Schematic representation of Tb–doped Y_2_O_3_ nanoparticle formation using salt-assisted FSP and subsequent water washing.

When a large amount of sodium nitrate was added to the yttrium and terbium nitrate precursors, the sodium salts melted as the flame was introduced, serving as molten salt. Y_2_O_3_ nucleated and fast grew in high crystallinity owing to the enhanced mass transfer in the presence of the molten salt. In addition, the excessive molten salts were considered to cover the surfaces of Y_2_O_3_ crystals, preventing them from making contact with other crystals. As the boiling point of sodium nitrate is approximately 380 °C [28], some of the sodium salts were lost. Therefore, the use of an excessive amount of sodium nitrate was critical in preventing Y_2_O_3_ crystals from aggregating. The possible route of nanoparticle generation using salt-assisted FSP is depicted in Figure 7. The sprayed droplets containing both metal precursors and sodium salts are dried by evaporating the solvents in the initial stage of FSP. The sodium salts melt and metal precursors decompose and oxidize to form oxide nucleates in the subsequent stage. Then, the nucleated particles undergo fast particle growth and aggregation to form highly crystalline particles embedded in the molten salt. The salts are removed during water washing, and the Y_2_O_3_ crystals are separated.

**Figure 8:**
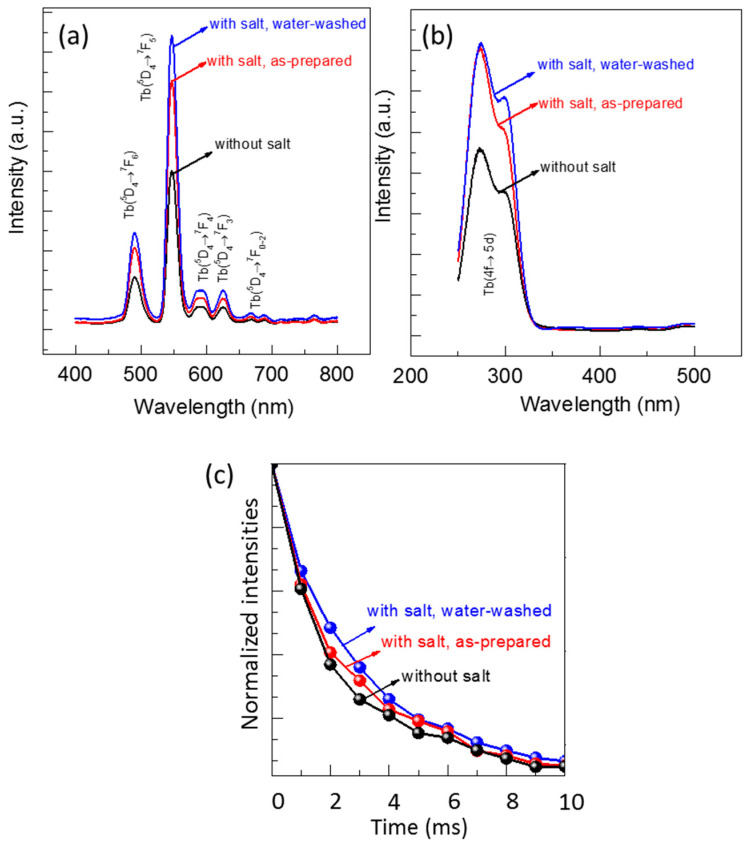
(**a**) Photoluminescence (PL) spectra for excitation at 365 nm; (**b**) photoluminescence excitation (PLE) spectra for emission at 547 nm; and (**c**) decay curves of emission at 547 nm and excitation at 300 nm.

The luminescent properties of the FSP products produced without and with sodium salts were evaluated using photoluminescence (PL) and photoluminescence excitation (PLE) spectra (Figure 8). A green light emission spanning from 480 to 700 nm was observed for all the samples when excited at 300 nm (Figure 8a). An intense peak appeared at 547 nm along with four other small peaks, which are the characteristic emissions of the Tb^3+^ ion. The intense peak corresponds to the transition from the ^5^D_4_ to ^7^F_5_ energy level, and small peaks at 492 nm, 590 nm, 625 nm, and 669 nm are associated with the transitions from ^5^D_4_ to ^7^F_6_, ^7^F_4_, ^7^F_3_, and ^7^F_2_ energy levels, respectively. A broad excitation peak at 250 to 330 nm was observed owing to the emission at 547 nm (Figure 8b), which was identified as a charge transfer peak from Y_2_O_3_ to Tb^3+^ ions [2].

The emission peak intensities at 547 nm of all the FSP products, shown in Figure 8a, are compared. The emission peak intensities of the products with sodium salt (both as-prepared and after water-washing) were higher than those of the products without sodium salts. The intensity of products with sodium salt slightly increased after washing with water, which was due to the removal of sodium impurities. Typically, as the particle size of phosphor decreases, additional surface defects arise, resulting in reduced luminescence. However, we discovered that the Y_2_O_3_:Tb^3+^ phosphors produced with sodium salts emit higher luminescence than those produced without the salts, even though their particle sizes are smaller. We considered that the high crystallinity and clean surfaces of Y_2_O_3_:Tb^3+^ nanoparticles, as observed using HR-TEM (Figure 4e), was attributed to the high luminescence. In addition, the incorporation of alkali ions into the crystal lattices of Y_2_O_3_:Tb^3+^, at either substitutional or interstitial sites, may have altered the crystal fields around the Tb^3+^ ions and charge transfer mechanisms, resulting in higher luminescence [29,30,31].

The luminescence decay curves of all the FSP products are obtained and compared as shown in Figure 8c. All the decay curves are well fitted to a single exponential function [17,18], and the calculated decay times are 2.21, 2.59, and 2.98 ms for Y_2_O_3_:Tb^3+^ produced without salts, Y_2_O_3_:Tb^3+^ produced with salts before washing, and Y_2_O_3_:Tb^3+^ produced with salts after washing, respectively. The increase in the decay time upon the addition of sodium salts is indicative of the charge transfer between the doped Na and Tb as well as reduced defect states in the Y_2_O_3_:Tb^3+^ crystals [29,32].

**Figure 9:**
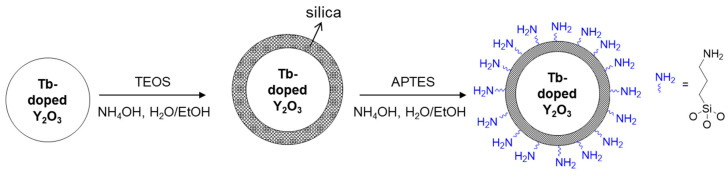
Schematics of silica shell formation and functionalization with aminopropyl groups on the surface of Y_2_O_3_:Tb^3+^ nanoparticles produced by salt-assisted FSP.

**Figure 10:**
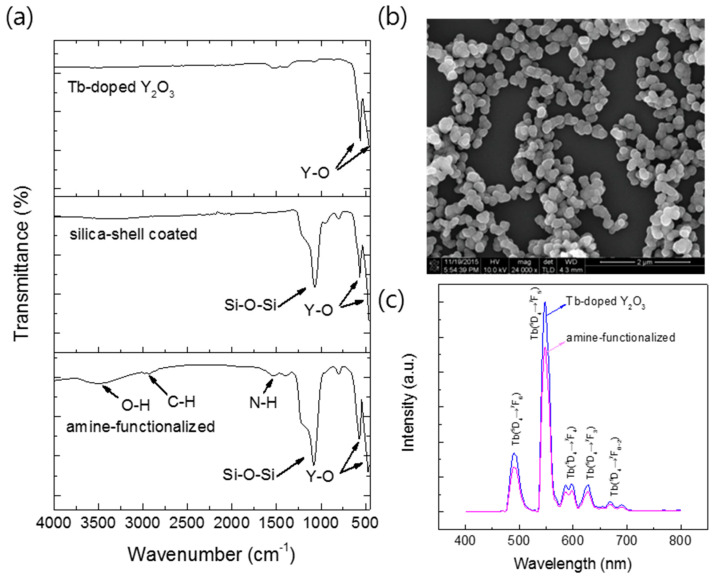
(**a**) Fourier transform infrared (FT-IR) transmittance spectra of Y_2_O_3_:Tb^3+^ nanoparticles prepared with salt and water-washing; Y_2_O_3_:Tb^3+^ nanoparticles coated with silica; Y_2_O_3_:Tb^3+^@silica particles functionalized with aminopropyl groups (from top to bottom); (**b**) SEM image of Y_2_O_3_:Tb^3+^@silica particles functionalized with aminopropyl groups; and (**c**) PL spectra for excitation at 365 nm of Y_2_O_3_:Tb^3+^ nanoparticles and Y_2_O_3_:Tb^3+^@silica particles functionalized with aminopropyl groups.

To examine the feasibility of the Y_2_O_3_:Tb^3+^ nanoparticles produced using the salt-assisted FSP for their application in bio-imaging, the particles were coated with silica, as it is a well-known biocompatible inorganic substance. The nanoparticles were first well dispersed in a solution using sonication, and a modified Stöber method [26] was then performed to form a silica shell on their surfaces (Figure 9, the first step). The Y_2_O_3_:Tb^3+^@silica core–shell particles were further functionalized with an organic compound, APTES, since amino-functional groups have been a great chemical source for coupling with bio-substances (Figure 9, the second step). The formation of the silica shell and ligand attachment were examined using FT-IR transmittance and SEM (Figure 10). The FT-IR spectrum of the silica coated Y_2_O_3_:Tb^3+^ exhibited a broad and strong peak corresponding to Si-O bonds at approximately 1070 cm^−1^ [33] along with peaks corresponding to Y_2_O_3_ at approximately 580 cm^−1^ and 496 cm^−1^ [34] (Figure 10a). The amine-functionalized Y_2_O_3_:Tb^3+^@silica particles revealed bands corresponding to C–H stretching modes at 2923 cm^−1^ and 2880 cm^−1^ [33], and NH_2_ deformation modes at approximately 1610 cm^−1^ [35], indicating the successful modification of Y_2_O_3_:Tb^3+^ nanoparticles with APTES. Besides those peaks, a broad peak corresponding to the O–H stretching mode was observed at around 3500 cm^−1^, which relates to the presence of water on the particle surface [35].

Changes of surface states were further confirmed by zeta-potential measurement [33]. Prior to the measurement, particles were well dispersed in a phosphate buffer (pH 7.4) to simulate the physiological condition for bio-imaging. The unmodified Y_2_O_3_:Tb^3+^ nanoparticles exhibited a negative potential, −5.6 mV, and the silica-coated Y_2_O_3_:Tb^3+^ nanoparticles showed a more negative potential, −19.5 mV, due to the presence of deprotonated oxide groups on the surface. However, a positive potential of +12.3 mV was observed with the APTES-coated Y_2_O_3_:Tb^3+^@silica particles, indicating the presence of NH_2_ groups on the surface [33]. The amount of APTES coated on the particles was measured by XRF and CHNS elemental analysis (Table 2). As a result, we found that N contents are 1.1 at %, which comprises approximately 1.77 mol % of yttrium (Y) in Y_2_O_3_. This is in accordance of the small bands of APTES in the FT-IR spectrum in Figure 10a.

**Figure 11:**
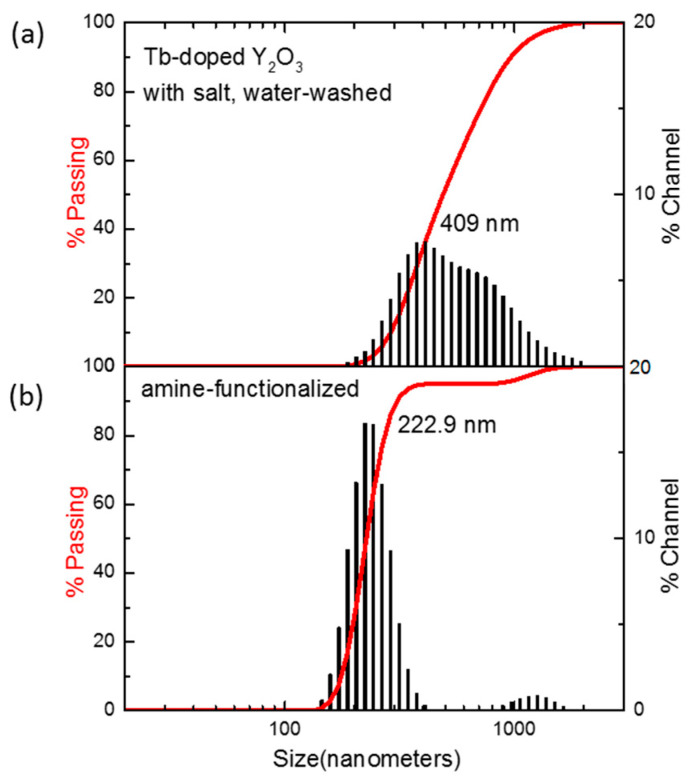
Particle size distributions of (**a**) Y_2_O_3_:Tb^3+^ nanoparticles prepared with salt and water washing; (**b**) Y_2_O_3_:Tb^3+^@silica particles functionalized with aminopropyl groups.

The SEM image of amine-functionalized Y_2_O_3_:Tb^3+^@silica particles exhibited well-dispersed particles of core–shell morphology (Figure 10b). The luminescence of the amine-functionalized particles was examined and compared with Y_2_O_3_:Tb^3+^ nanoparticles without a surface coating, which showed a slight reduction in intensity after surface functionalization (Figure 10c). The reduction originates from the dilution effect of phosphors, which are responsible for luminescence, due to the addition of luminescence-free silica and organic functional groups. Nevertheless, the phosphors functionalized with silica and amino groups exhibited higher dispersity in an aqueous solution compared with that of bare phosphors, which was confirmed by a dynamic light scattering analysis of unmodified particles and amine-functionalized Y_2_O_3_:Tb^3+^@silica particles dispersed in a phosphate buffer (Figure 11). The size distribution of unmodified particles was found to be somewhat broad (average size = 409 nm) (Figure 11a), which is ascribed to the aggregation of unmodified particles, since it is well known that nanoparticles as small as 100 nm have a high surface energy so that they tend to aggregate by themselves [36]. However, the much smaller average size of amino-functionalized particles (223 nm) was observed (Figure 11b), although the size of particles became almost twice larger than the unmodified ones due to the presence of a silica shell according to the SEM image in Figure 10b. The enhanced dispersity originates from aminopropyl ligands anchored on particle surface, which have repulsive interactions due to the Van der Waals force of organic moiety and positive charge of amino groups.

## 4. Conclusion

We successfully modified FSP for the synthesis of Y_2_O_3_:Tb^3+^ nanophosphor by adding sodium salt, followed by washing with water. The Y_2_O_3_:Tb^3+^ nanoparticles generated were much less aggregated than those produced without sodium salt, and they exhibited higher crystallinity and luminescence. This salt-assisted FSP was particularly attractive not only because it can be adopted for a continuous reaction to produce nanoparticles in a large scale but also because the resulting products have high crystallinity and luminescence without post-annealing. The surface functionalization of the Y_2_O_3_:Tb^3+^ nanoparticles with the silica shell and organic ligands was also successfully demonstrated by FT-IR and surface potentials. The loading quantity of amino functional groups on the Y_2_O_3_:Tb^3+^ nanophosphor was measured to be 1.7 at % of yttrium by XRF and CHNS analysis. The high dispersity of the amine-modified nanophosphors was observed by DLS analysis in a physiological condition (pH 7.4), proving that they are highly applicable for bio-imaging.

## Figures and Tables

**Table 1:** Gas compositions of different flame conditions.

No. run	#1	#2	#3	#4	#5	#6	#7
CH_4_ (L/min)	6	6	6	9	9	9	9
O_2_ (L/min)	0	6	12	0	6	12	18
Air (L/min)	6	6	6	6	6	6	6
Max. Temperature (°C)	850	775	700	975	940	1050	1150

**Table 2:** Elemental compositions of Y_2_O_3_:Tb^3+^@silica particles functionalized with aminopropyl groups by X-ray fluorescence (XRF) and CHNS analysis.

Element	Y	Tb	Na	Si	N	C
at %	61.8	1.0	0.4	29.9	1.1	5.8
